# Paraneoplastic Myopathy as a Possible Initial Presentation of Sporadic Burkitt Lymphoma in an Immunocompetent Adult: A Case Report

**DOI:** 10.1002/cnr2.70669

**Published:** 2026-09-01

**Authors:** Abolfazl Khalafi‐Nezhad, Dena Firouzabadi, Vahid mohammadkarimi, Amirreza Dehghanian

**Affiliations:** ^1^ Hematology Research Center, Department of Hematology, Medical Oncology and Stem Cell Transplantation Shiraz University of Medical Sciences Shiraz Iran; ^2^ Department of Clinical Pharmacy, School of Pharmacy Shiraz University of Medical Sciences Shiraz Iran; ^3^ Molecular Pathology and Cytogenetics Section, Department of Pathology, School of Medicine Shiraz University of Medical Sciences Shiraz Iran

**Keywords:** Burkitt's lymphoma, creatine phosphokinase, extranodal lymphoma, hyper‐CVAD chemotherapy, paraneoplastic myopathy

## Abstract

**Background:**

Burkitt's lymphoma (BL) is a highly aggressive B‐cell non‐Hodgkin lymphoma with rapid proliferation and early systemic dissemination. Neuromuscular manifestations are rare and are typically secondary to direct infiltration or metabolic complications. Paraneoplastic myopathy as the initial presentation of BL is exceedingly uncommon, particularly in immunocompetent adults.

**Case Presentation:**

We report the case of an immunocompetent adult male who presented with acute progressive proximal muscle weakness and markedly elevated creatine phosphokinase (CPK) levels. Extensive autoimmune, metabolic, and endocrine evaluations were unremarkable. Electrophysiological studies supported a primary myopathic process. Contrast‐enhanced computed tomography revealed left axillary lymphadenopathy and splenomegaly without generalized lymphadenopathy. Excisional lymph node biopsy confirmed sporadic Burkitt's lymphoma with MYC rearrangement. Muscle biopsy was deferred due to rapid disease progression and the urgent need for oncologic treatment. The patient was treated with the Hyper‐CVAD regimen combined with rituximab and central nervous system prophylaxis. Following chemotherapy, muscle strength improved rapidly, accompanied by a marked decline in serum CPK levels.

**Conclusion:**

This case highlights a rare paraneoplastic manifestation of Burkitt's lymphoma presenting as acute myopathy. Rapid clinical and biochemical response to chemotherapy supports a tumor‐mediated mechanism. Clinicians should consider underlying hematologic malignancy in patients with unexplained myopathy and elevated muscle enzymes, as early diagnosis and prompt treatment may significantly improve outcomes.

## 1 Introduction

Burkitt's lymphoma (BL) is a highly aggressive B‐cell non‐Hodgkin lymphoma characterized by MYC gene rearrangement and an exceptionally high proliferative index. Although it predominantly affects children and young adults, sporadic BL increasingly presents in immunocompetent adults, particularly males over 40 years of age [1]. The clinical manifestations of BL are largely determined by the sites of extranodal involvement, most commonly the abdomen, bone marrow, and central nervous system [2, 3].

Neurological and neuromuscular manifestations of BL are uncommon and are generally attributed to direct tumor infiltration, metabolic complications, or treatment‐related toxicity. Previously reported neurological presentations have included paraplegia, cranial neuropathies, Guillain–Barré syndrome, and other manifestations related to direct nervous system involvement [4, 5, 6, 7]. Inflammatory and paraneoplastic myopathies have also been described in association with lymphoma more broadly [8]; however, clinically overt paraneoplastic myopathy preceding the diagnosis of sporadic BL in an immunocompetent adult appears to be exceptionally rare.

Herein, we report the case of a 42‐year‐old immunocompetent man who initially presented with progressive proximal muscle weakness, myalgia, and markedly elevated serum creatine phosphokinase (CPK) levels before the diagnosis of sporadic BL was established. Comprehensive clinical, laboratory, electrophysiological, and staging investigations excluded more common causes of neuromuscular dysfunction, including direct central nervous system involvement. Furthermore, the patient's rapid clinical recovery, progressive normalization of serum CPK levels, and complete resolution of the electrodiagnostic abnormalities following lymphoma‐directed chemotherapy strongly supported the diagnosis of a probable paraneoplastic myopathy. This case highlights the importance of considering an occult hematologic malignancy in the differential diagnosis of otherwise unexplained proximal myopathy and emphasizes the value of a multidisciplinary diagnostic approach in recognizing rare paraneoplastic presentations of aggressive lymphomas.

## 2 Case Presentation

A 42‐year‐old previously healthy and immunocompetent man presented to Namazi Hospital, Shiraz, Iran, in March 2023 with a short history of progressively worsening proximal muscle weakness, predominantly involving the lower extremities. He also reported diffuse myalgia, increasing difficulty rising from a seated position, and difficulty climbing stairs. There was no history of recent infection, trauma, strenuous physical activity, toxin exposure, alcohol or illicit drug use, or treatment with statins or other potentially myotoxic medications. He had no significant medical or surgical history and no known family history of neuromuscular disorders or hematologic malignancies. He denied numbness, paresthesia, radicular pain, bowel or bladder dysfunction, and other sensory complaints. The patient had not received corticosteroids or any other medications known to cause myopathy before the onset of muscle weakness or prior to the initial electrodiagnostic evaluation. Corticosteroids were initiated only after the diagnosis of BL as part of the Hyper‐CVAD treatment regimen.

On admission, the patient was fully conscious, alert, and oriented. Neurological examination demonstrated symmetric proximal muscle weakness involving both upper and lower extremities, with a Medical Research Council (MRC) muscle strength Grade of 3/5, indicating active movement against gravity but not against resistance. Deep tendon reflexes were diffusely decreased (1+ bilaterally). Cranial nerve examination was normal, sensory examination was unremarkable, and sphincter function was intact. No cerebellar deficits or pathological reflexes were observed.

Laboratory evaluation revealed an elevated serum CPK level of 900 IU/L (reference range, 24–195 IU/L), raising concern for an underlying myopathic process. Autoimmune evaluation including antinuclear antibody (ANA), myositis‐specific antibodies (anti–Jo‐1, anti–Mi‐2, anti–SRP, and anti–HMGCR), as well as thyroid function tests (TSH, free T4, free T3) and thyroid autoantibodies (anti‐TPO and anti‐thyroglobulin) were all within normal limits, effectively excluding immune‐mediated and endocrine‐related myopathies (Table 1). As part of the diagnostic evaluation, HIV serology was negative. EBV serology was consistent with previous EBV infection (positive EBV VCA IgG with negative IgM).

**TABLE 1:** Laboratory and biochemical findings.

Parameter	Result	Reference range
Hemoglobin (g/dL)	10.8	12–16
WBC (× 10^9^/L)	8.2	4–10
Platelet count (× 10^9^/L)	240	150–400
ESR (mm/h)	48	< 20
LDH (U/L)	1280	100–250
Uric acid (mg/dL)	9.8	3.5–7.2
Creatinine (mg/dL)	1.4	0.6–1.2
AST (U/L)	52	< 35
ALT (U/L)	60	< 45
CPK (U/L)	900	30–200
Calcium (mg/dL)	8.9	8.5–10.5
Serum potassium (mEq/L)	5.4	3.5–5.1
EBV IgM/IgG	Negative/Positive	—
HIV, HBV, HCV	Negative	—
TSH (mIU/L)	1.2	0.4–4.5
Free T4 (ng/dL)	1.1	0.8–1.8
Free T3 (pg/mL)	3	2.3–4.2
Anti‐thyroid peroxidase antibody (IU/mL)	7	< 35
Antinuclear Antibody (ANA)	Negative	—
Anti–Jo‐1 (Histidyl‐tRNA synthetase)	Negative	—
Anti–Mi‐2	Negative	—
Anti–Signal Recognition Particle (Anti‐SRP)	Negative	—
Anti–HMG‐CoA Reductase (Anti‐HMGCR)	Negative	—

Electromyography (EMG) and Nerve Conduction Study (NCS) demonstrated a predominantly myopathic pattern characterized by short‐duration, low‐amplitude motor unit potentials with early recruitment, consistent with an inflammatory or necrotizing myopathy. Mild secondary axonal features were noted; however, the overall findings favored a primary myopathic process rather than a neuropathic disorder (Table 2).

**TABLE 2:** Electromyography (EMG) and nerve conduction study (NCS) findings before and after treatment.

Test	Muscle/nerve	Pre‐treatment findings	Post‐treatment findings	Interpretation
EMG	Left First Dorsal Interosseous	No spontaneous activity; ↓ MUAP amplitude; ↓ MUAP duration; early recruitment	No spontaneous activity; normal MUAP amplitude and duration; normal recruitment	Myopathic → Normal
	Left Deltoid	Fibrillation +++; PSW +++; ↓ MUAP amplitude; ↓ MUAP duration; early recruitment	No fibrillation; no PSWs; normal MUAP amplitude and duration; normal recruitment	Myopathic → Normal
	Left Tensor Fascia Lata	Fibrillation +++; PSW +++; CRD+; ↓ MUAP amplitude; ↓ MUAP duration; early recruitment	No abnormal spontaneous activity; normal MUAP amplitude and duration; normal recruitment	Myopathic → Normal
Sensory NCS	Right Median (Wrist–Digit III)	Latency 4.0 ms; Amplitude 24.0 μV	Normal (unchanged)	Normal
	Left Ulnar (Wrist–Digit V)	Latency 3.4 ms; Amplitude 27.7 μV	Normal (unchanged)	Normal
	Left Sural (Calf–Lat. Malleolus)	Latency 2.55 ms; Amplitude 22.1 μV	Normal (unchanged)	Normal
Motor NCS	Left Median–APB	Latency 3.9 ms; Amplitude 6.4 mV	Normal (unchanged)	Normal
	Right Ulnar–ADM	Latency 3.5–7.95 ms; Amplitude 3.4–3.2 mV; Velocity 47.2 m/s	Normal (unchanged)	Normal
	Left Common Peroneal–EDB	Latency 5.8–12.45 ms; Amplitude 2.2–2.1 mV; Velocity 46.6 m/s	Normal (unchanged)	Normal
	Right Tibial–AH	Latency 5.6 ms; Amplitude 3.7 mV	Normal (unchanged)	Normal

Given the acute presentation and biochemical evidence of muscle injury, the differential diagnosis initially included inflammatory myopathy, paraneoplastic myopathy, metabolic myopathy, and less likely infectious or toxic etiologies.

**FIGURE 1:**
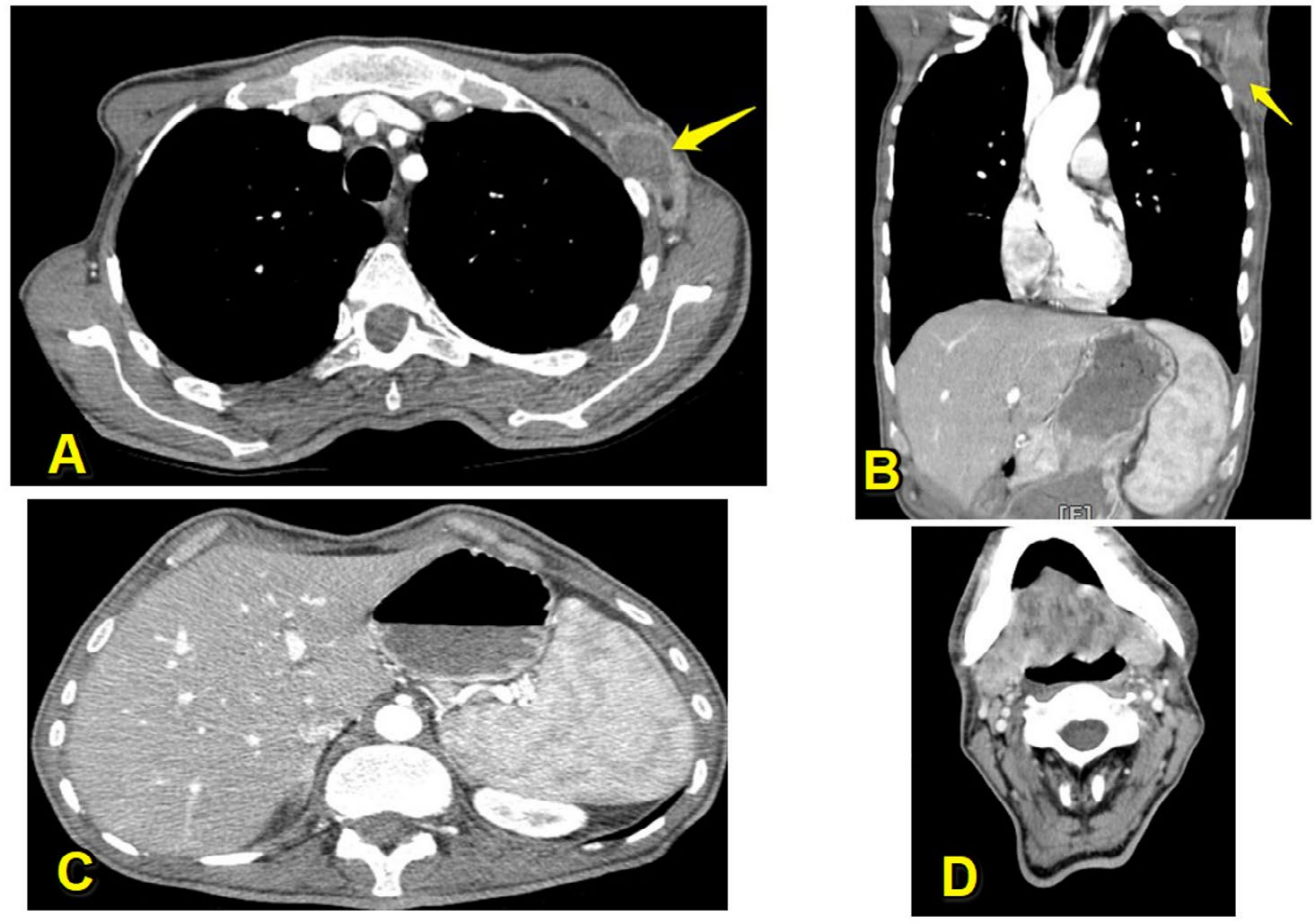
Contrast‐enhanced computed tomography (CT) findings obtained during the initial diagnostic evaluation. (A) Axial contrast‐enhanced chest CT demonstrates focal soft‐tissue thickening along the left chest wall (arrow). (B) Coronal reconstructed chest CT shows longitudinal extension of the chest wall lesion (arrow) without evidence of pulmonary parenchymal involvement. (C) Axial contrast‐enhanced abdominal CT reveals splenomegaly without focal hepatic or splenic lesions. (D) Axial contrast‐enhanced neck CT demonstrates no discrete cervical mass or pathological cervical lymphadenopathy. Collectively, these imaging findings suggested a systemic lymphoproliferative process and prompted further diagnostic evaluation, including excisional biopsy of the left axillary lymph node, which established the diagnosis of sporadic Burkitt's lymphoma.

During further evaluation, contrast‐enhanced cross‐sectional imaging (CT scan) demonstrated enlarged left axillary lymph nodes, measuring 3.5 × 2.4 cm. Splenomegaly was also present, without focal hepatic or splenic lesions (Figure 1). No definite mass lesions were identified in the neck, chest, abdomen, or pelvis.

In view of these imaging findings and the clinical suspicion of an underlying lymphoproliferative disorder, an excisional biopsy of the axillary lymph node was prioritized to establish a definitive diagnosis.

**FIGURE 2:**
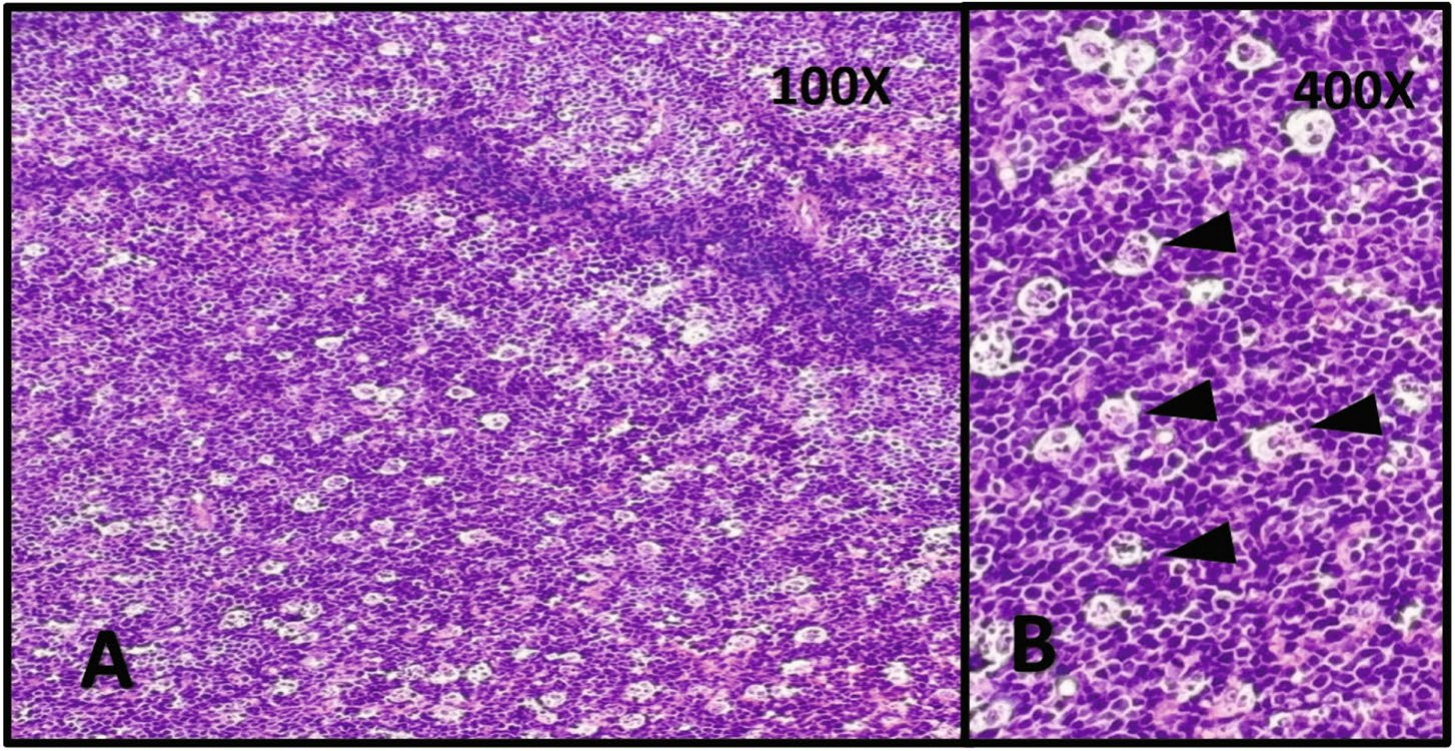
Histopathological findings of the excised lymph node. (A, B) Hematoxylin and eosin‐stained sections show complete effacement of the nodal architecture by a diffuse proliferation of monotonous medium‐sized lymphoid cells. The neoplastic cells exhibit round to slightly irregular nuclei, coarse to clumped chromatin, multiple small nucleoli, and scant to moderately basophilic cytoplasm. Numerous mitotic figures and apoptotic bodies, together with abundant karyorrhectic debris, are present throughout the tumor. Frequent tingible‐body macrophages (arrowheads) containing apoptotic debris create the characteristic “starry‐sky” appearance, a hallmark histopathological feature of Burkitt's lymphoma. Original magnification: (A) × 100; (B) × 400.

In April 2023, histopathological examination of the excised axillary lymph node demonstrated diffuse effacement of the nodal architecture by a monomorphic proliferation of medium‐sized atypical lymphoid cells with frequent mitotic figures and a characteristic starry‐sky appearance (Figure 2). Immunohistochemistry demonstrated positivity for CD20, CD10, and BCL6, with a Ki‐67 proliferation index approaching 100%, consistent with BL. Cytogenetic analysis demonstrated MYC gene rearrangement. Together with the histomorphological and immunophenotypic findings, these results strongly supported the diagnosis of sporadic BL.

The available histomorphological, immunohistochemical, and cytogenetic findings were considered strongly supportive of BL; however, the absence of a broader immunohistochemical panel represents a limitation of the diagnostic assessment.

Following histopathological confirmation of the diagnosis, staging evaluation was performed in April 2023. Bone marrow aspiration and biopsy showed no evidence of lymphoma involvement. Baseline magnetic resonance imaging of the brain and cervical, thoracic, and lumbar spine showed no evidence of intracranial, spinal cord, nerve root, or leptomeningeal involvement. Cerebrospinal fluid cytological examination was negative for malignant cells. Positron emission tomography/computed tomography (PET/CT) was not performed. Based on axillary lymph node involvement and splenomegaly on computed tomography, in the absence of bone marrow or central nervous system involvement, the patient was classified as having Ann Arbor Stage III BL.

Given the aggressive and rapidly progressive nature of BL and the urgent need to initiate chemotherapy, muscle biopsy was deferred because its findings were unlikely to alter the immediate therapeutic approach and the procedure could have delayed potentially life‐saving treatment. The subsequent rapid improvement in muscle strength and normalization of serum CPK following lymphoma‐directed chemotherapy further supported, although did not definitively confirm, a paraneoplastic etiology.

Later in April 2023, the patient was started on intensive combination chemotherapy using the Hyper‐CVAD regimen, consisting of alternating cycles of cyclophosphamide, vincristine, doxorubicin, and dexamethasone (Course A) and high‐dose methotrexate with cytarabine (Course B). Because the lymphoma cells expressed CD20, rituximab was incorporated into the treatment regimen, and standard central nervous system prophylaxis was administered according to institutional protocols.

**FIGURE 3:**
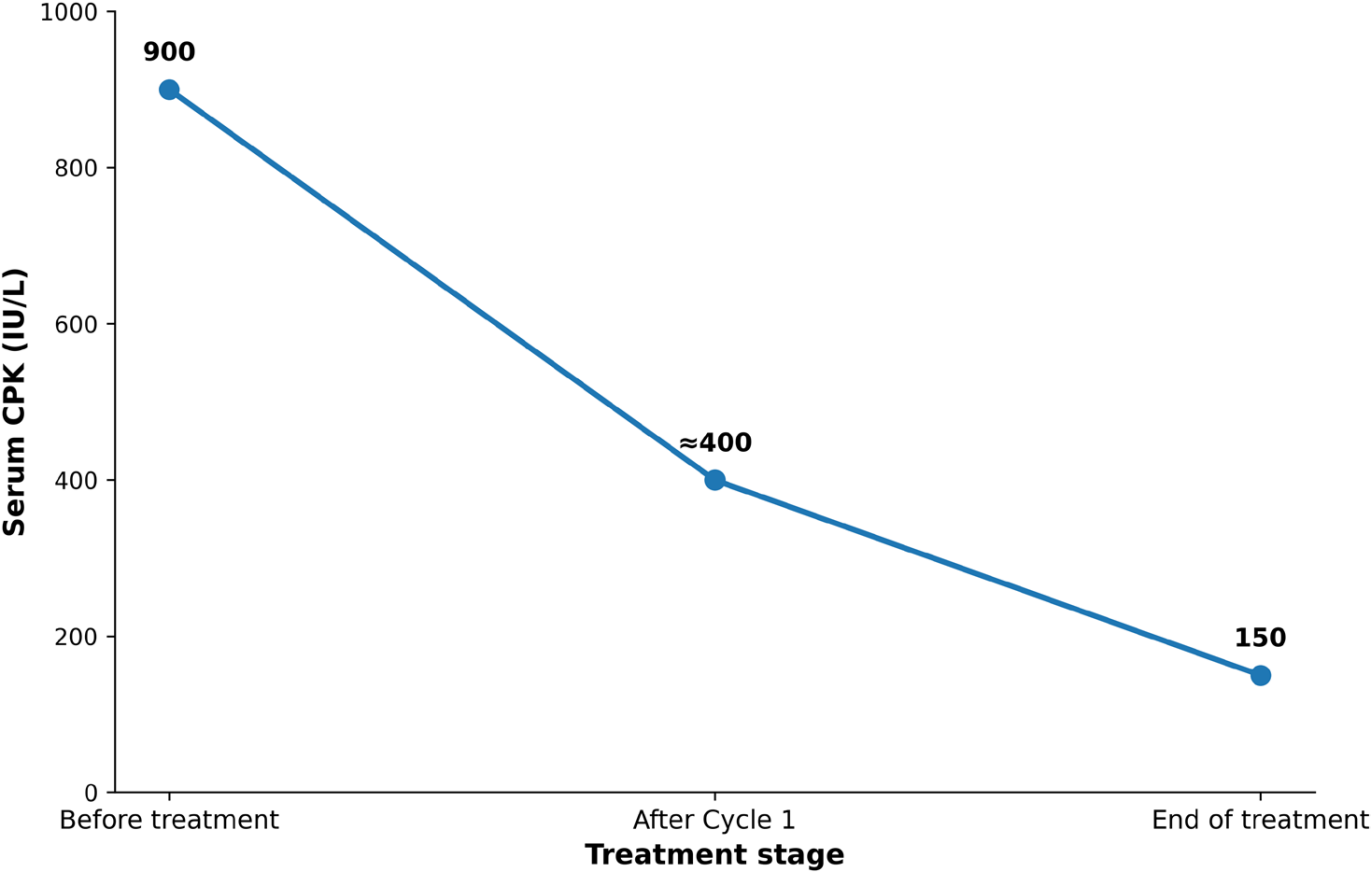
Serial serum creatine phosphokinase (CPK) levels during lymphoma‐directed treatment. Serum CPK was elevated to 900 IU/L before initiation of chemotherapy, decreased to approximately 400 IU/L after completion of the first Hyper‐CVAD cycle, and further declined to 150 IU/L following completion of the full treatment course. The progressive biochemical improvement occurred in parallel with the patient's clinical recovery and resolution of the previously documented myopathic changes on follow‐up electromyography. CPK, creatine phosphokinase; Hyper‐CVAD, hyperfractionated cyclophosphamide, vincristine, doxorubicin, and dexamethasone alternating with high‐dose methotrexate and cytarabine.

By May 2023, after completion of the first Hyper‐CVAD cycle, the patient exhibited rapid and marked improvement in muscle strength, with serum CPK decreasing from 900 IU/L before treatment to approximately 400 IU/L. By August 2023, following completion of the full treatment course, serum CPK had further declined to 150 IU/L, accompanied by sustained clinical improvement (Figure 3).

In September 2023, follow‐up electromyography and nerve conduction studies demonstrated complete resolution of the previously documented myopathic abnormalities, including normalization of spontaneous activity, motor unit morphology, and recruitment patterns. Both the baseline and follow‐up electrodiagnostic studies were independently reviewed by an experienced neurologist, who confirmed complete resolution of the initial myopathic abnormalities following chemotherapy (Table 2).

At the most recent clinical follow‐up in March 2026, the patient remained asymptomatic, with normal muscle strength and no recurrence of myopathic symptoms.

Taken together, the clinical course, biochemical response, and normalization of the electrodiagnostic abnormalities strongly favored a probable paraneoplastic myopathy associated with BL.

No treatment‐related adverse events or unexpected complications were observed during hospitalization or after initiation of Hyper‐CVAD chemotherapy. A chronological summary of the patient's clinical course, including the diagnostic workup, staging evaluation, treatment, and clinical outcome, is provided in Table 3.

**TABLE 3:** Chronological timeline of the patient's clinical course, diagnostic evaluation, treatment, and follow‐up.

Month/Year	Clinical phase/Milestone	Clinical events and findings
March 2023	Initial presentation	Progressive proximal muscle weakness, diffuse myalgia, and difficulty climbing stairs and rising from a seated position.
March 2023	Initial neurological assessment	Symmetric proximal muscle weakness involving the upper and lower extremities (MRC Grade 3/5), diminished deep tendon reflexes (1+), intact cranial nerves, preserved sensation, and normal sphincter function.
March 2023	Initial laboratory evaluation	Serum CPK was elevated to 900 IU/L. Autoimmune, endocrine, infectious, metabolic, and toxic causes of myopathy were investigated and no alternative etiology was identified.
March 2023	Electrodiagnostic evaluation	EMG/NCS demonstrated a predominantly myopathic pattern with short‐duration, low‐amplitude motor unit potentials and early recruitment.
March 2023	Imaging studies	Contrast‐enhanced CT demonstrated left axillary lymphadenopathy and splenomegaly, raising suspicion for an underlying lymphoproliferative disorder.
April 2023	Histopathological diagnosis	Excisional biopsy of the left axillary lymph node demonstrated morphological and immunophenotypic findings strongly supportive of sporadic Burkitt lymphoma; MYC rearrangement was detected.
April 2023	Staging evaluation	Bone marrow aspiration and biopsy showed no lymphoma involvement. Brain and cervical/thoracic/lumbar spine MRI showed no CNS involvement, and CSF cytology was negative for malignant cells. PET/CT was not performed. The disease was classified as Ann Arbor Stage III.
April 2023	Treatment initiation	Hyper‐CVAD chemotherapy combined with rituximab and CNS prophylaxis was initiated promptly because of the aggressive nature of Burkitt lymphoma.
May 2023	Early treatment response – after Cycle 1	Muscle strength improved markedly, and serum CPK decreased from 900 IU/L to approximately 400 IU/L after the first Hyper‐CVAD cycle.
August 2023	Completion of treatment	Following completion of the full lymphoma‐directed treatment course, serum CPK further decreased to 150 IU/L, accompanied by sustained clinical improvement.
September 2023	Electrophysiological follow‐up	Follow‐up EMG/NCS demonstrated complete resolution of the previously documented myopathic abnormalities, with normalization of spontaneous activity, motor unit morphology, and recruitment patterns.
March 2026	Last clinical follow‐up	The patient remained asymptomatic, with normal muscle strength, no recurrence of myopathic symptoms

## 3 Discussion

BL is a highly aggressive mature B‐cell non‐Hodgkin lymphoma characterized by an exceptionally high proliferative rate and rapid clinical progression. Although extranodal involvement is common, neurological manifestations as the presenting feature are uncommon and usually result from direct involvement of the central or peripheral nervous system rather than an indirect immune‐mediated mechanism [9]. The present case is clinically distinctive because progressive proximal myopathy preceded the diagnosis of sporadic BL in an immunocompetent adult, creating a substantial diagnostic challenge before the underlying malignancy was identified.

The most significant finding of this case was the occurrence of progressive proximal muscle weakness accompanied by markedly elevated serum CPK levels and electromyographic findings consistent with a primary myopathic process before the diagnosis of lymphoma. Because muscle weakness was the dominant presenting symptom, the initial differential diagnosis included inflammatory myopathies, endocrine and metabolic disorders, infectious myositis, toxic myopathy, and paraneoplastic neuromuscular syndromes. The absence of myositis‐specific autoantibodies, normal endocrine investigations, and the lack of clinical evidence supporting alternative causes narrowed the differential diagnosis but did not establish a definitive etiology. Consequently, the underlying hematologic malignancy might easily have been overlooked if systemic evaluation had not been pursued.

Neurological complications associated with BL have been described previously; however, the reported manifestations differ considerably from those observed in the present patient. Turgut et al. described a child presenting with paraplegia caused by spinal epidural BL, whereas Bouattour et al. reported Guillain–Barré syndrome occurring in association with sporadic BL [4, 5]. Other reported neurological presentations have included bilateral numb chin syndrome and isolated oculomotor nerve palsy, both of which reflected cranial nerve or meningeal involvement rather than primary skeletal muscle disease [6, 7]. In contrast, our patient demonstrated symmetrical proximal muscle weakness without cranial neuropathy, sensory impairment, sphincter dysfunction, cerebrospinal fluid involvement, or bone marrow infiltration. Electromyography supported a predominantly myopathic rather than neuropathic process, distinguishing this presentation from previously reported neurological manifestations of BL.

Furthermore, unlike many previously published reports in which neurological symptoms developed concurrently with advanced lymphoma or resulted from direct tumor infiltration, the neuromuscular manifestations in the present case preceded the diagnosis of lymphoma and represented the principal reason for medical evaluation. This unusual clinical sequence highlights the importance of considering an underlying hematologic malignancy in patients with otherwise unexplained proximal myopathy, particularly when routine investigations fail to identify an autoimmune, endocrine, metabolic, or infectious cause. Early recognition of this association may facilitate prompt diagnosis and timely initiation of definitive lymphoma‐directed therapy.

Paraneoplastic myopathies are more commonly associated with solid malignancies and selected hematologic neoplasms, particularly Hodgkin lymphoma and intravascular large B‐cell lymphoma, whereas their occurrence in BL is exceptionally uncommon. Proposed pathogenic mechanisms include immune‐mediated muscle injury, cytokine‐driven inflammation, molecular mimicry between tumor antigens and skeletal muscle proteins, and dysregulated immune responses triggered by the underlying malignancy [10, 11]. These mechanisms may result in inflammatory or necrotizing muscle injury before the appearance of constitutional or tumor‐related symptoms, making early diagnosis particularly challenging.

Although muscle biopsy remains the gold standard for establishing the pathological subtype of inflammatory myopathy and is generally recommended in patients with unexplained muscle weakness [12], it was not performed in the present case because of the aggressive clinical course of BL and the urgent need to initiate definitive chemotherapy. Given the rapidly progressive nature of this disease, delaying treatment to obtain histopathological confirmation of muscle involvement was considered unlikely to alter immediate management and might have postponed potentially life‐saving therapy. Consequently, the diagnosis of probable paraneoplastic myopathy was based on the integration of clinical findings, electrophysiological evidence, exclusion of alternative etiologies, and subsequent therapeutic response rather than histological confirmation.

A notable finding in the present case was the rapid parallel improvement in both neurological symptoms and biochemical markers following lymphoma‐directed treatment. Muscle strength improved soon after chemotherapy, accompanied by a marked decline in serum CPK levels from 900 IU/L to 150 IU/L. This close temporal relationship strongly supports a tumor‐associated mechanism underlying the myopathy and is consistent with observations in other paraneoplastic neuromuscular syndromes, in which successful treatment of the underlying malignancy frequently results in improvement of neurological manifestations [13]. Nevertheless, therapeutic response alone cannot definitively establish a paraneoplastic diagnosis, and the absence of muscle biopsy remains an important limitation that should be acknowledged when interpreting the findings.

Several mechanisms may explain this uncommon presentation. Experimental and clinical studies suggest that aggressive B‐cell lymphomas can induce skeletal muscle injury through excessive cytokine production, immune dysregulation, or cross‐reactivity between tumor‐associated antigens and muscle fibers [14]. Although direct evidence for these mechanisms in BL remains limited, the absence of central nervous system involvement, negative cerebrospinal fluid cytology, negative bone marrow examination, exclusion of alternative causes of myopathy, and rapid resolution following lymphoma‐directed therapy collectively favor an indirect immune‐mediated process rather than direct tumor infiltration of skeletal muscle.

Corticosteroid‐induced myopathy represents an important differential diagnosis in patients with lymphoma. However, this explanation was considered unlikely in the present case because the muscle weakness, elevated serum CPK level, and myopathic electrodiagnostic abnormalities were documented before the initiation of corticosteroid‐containing chemotherapy. Corticosteroids were administered only after the diagnosis of BL as part of the Hyper‐CVAD regimen. Moreover, the patient's muscle strength, serum CPK level, and electrodiagnostic abnormalities improved despite subsequent corticosteroid exposure, further arguing against steroid‐induced myopathy and supporting the diagnosis of probable paraneoplastic myopathy.

Another important observation in the present case was the favorable clinical response following prompt initiation of lymphoma‐directed chemotherapy. BL is characterized by an extremely rapid doubling time and requires immediate treatment with intensive multi‐agent chemotherapy regimens, such as Hyper‐CVAD combined with central nervous system prophylaxis, to improve survival outcomes [15]. In our patient, systemic chemotherapy not only controlled the underlying malignancy but was also followed by rapid improvement in proximal muscle strength and normalization of serum CPK levels. Although this temporal association does not establish causality, it is consistent with the hypothesis that the neuromuscular manifestations were closely related to the underlying lymphoma rather than representing an independent primary muscle disorder.

This case also highlights several important clinical implications. First, proximal muscle weakness with elevated serum CPK should not automatically be attributed to primary inflammatory myopathy, particularly when conventional diagnostic investigations are inconclusive. Careful systemic evaluation, including assessment for occult malignancy, may be warranted in selected patients with atypical clinical features. Second, early recognition of the underlying lymphoma is critical because BL is a rapidly progressive but highly chemosensitive malignancy, and delays in diagnosis may adversely affect both neurological recovery and overall prognosis. Finally, this case emphasizes the importance of multidisciplinary collaboration among neurologists, hematologists, pathologists, and oncologists when evaluating patients with unexplained myopathy and suspected paraneoplastic syndromes.

The principal limitation of this report is the absence of histopathological confirmation by muscle biopsy. Consequently, the diagnosis of probable paraneoplastic myopathy remains based on clinical presentation, electrophysiological findings, exclusion of alternative etiologies, and the marked clinical and biochemical improvement observed after lymphoma‐directed chemotherapy. In addition, the nature of a single case report precludes establishing a causal relationship or determining the true frequency of this uncommon presentation. Nevertheless, the detailed clinical evaluation, comprehensive exclusion of other causes of myopathy, and consistent therapeutic response provide evidence supporting a likely association between BL and the patient's neuromuscular manifestations.

A further limitation of this report is the restricted immunohistochemical work‐up of the lymph‐node specimen. Although the characteristic morphology, germinal‐center B‐cell phenotype, nearly 100% Ki‐67 proliferation index, and MYC rearrangement strongly supported BL, additional markers such as BCL2, T‐cell lineage markers, ALK, and EBER in situ hybridization were not assessed. Consequently, the pathological diagnosis should be interpreted in the context of the available integrated findings rather than as being based on a fully comprehensive immunophenotypic panel.

## 4 Conclusion

In conclusion, this case highlights an unusual presentation of sporadic BL in an immunocompetent adult, in which progressive proximal myopathy preceded the diagnosis of the underlying malignancy. Although histopathological confirmation of muscle involvement was unavailable, the combination of clinical findings, electrophysiological evidence, exclusion of alternative etiologies, and rapid improvement following lymphoma‐directed chemotherapy strongly supports a probable paraneoplastic mechanism. This case underscores the importance of considering occult hematologic malignancies in the differential diagnosis of otherwise unexplained proximal myopathy, particularly when routine investigations are inconclusive. Early recognition of this uncommon presentation may facilitate timely diagnosis, prompt initiation of appropriate treatment, and improved clinical outcomes.

## Author Contributions

**Abolfazl Khalafi‐Nezhad:** conceptualization, investigation, methodology, validation, visualization, writing – review and editing, supervision, writing – original draft, formal analysis. **Dena Firouzabadi:** investigation, methodology, validation, visualization, writing – review and editing, formal analysis, writing – original draft. **Vahid mohammadkarimi:** investigation, funding acquisition, writing – original draft, visualization, methodology, validation, formal analysis, data curation. **Amirreza Dehghanian:** investigation, funding acquisition, writing – original draft, visualization, validation, methodology, supervision, project administration.

## Funding

The authors have nothing to report.

## Ethics Statement

The current study was approved by the Ethical Committee of Shiraz University of Medical Sciences (IR.SUMS.MED.REC.1404.473). Written informed consent was obtained from the patient for publication of this Case Report and all related clinical, radiologic, and pathologic images. A copy of the signed consent form is available for review by the journal editor upon request.

## Conflicts of Interest

The authors declare no conflicts of interest.

## Data Availability

The data that support the findings of this study are available on request from the corresponding author. The data are not publicly available due to privacy or ethical restrictions.
